# Speaker-Sex Discrimination for Voiced and Whispered Vowels at Short Durations

**DOI:** 10.1177/2041669516671320

**Published:** 2016-10-03

**Authors:** David R. R. Smith

**Affiliations:** University of Hull, UK

**Keywords:** Speaker-sex discrimination, speech, voiced, whispered, duration, vocal-tract length, pitch

## Abstract

Whispered vowels, produced with no vocal fold vibration, lack the periodic temporal fine structure which in voiced vowels underlies the perceptual attribute of pitch (a salient auditory cue to speaker sex). Voiced vowels possess no temporal fine structure at very short durations (below two glottal cycles). The prediction was that speaker-sex discrimination performance for whispered and voiced vowels would be similar for very short durations but, as stimulus duration increases, voiced vowel performance would improve relative to whispered vowel performance as pitch information becomes available. This pattern of results was shown for women’s but not for men’s voices. A whispered vowel needs to have a duration three times longer than a voiced vowel before listeners can reliably tell whether it’s spoken by a man or woman (∼30 ms vs. ∼10 ms). Listeners were half as sensitive to information about speaker-sex when it is carried by whispered compared with voiced vowels.

## Introduction

The world is full of complex dynamically changing sources of sound. One source of sound is other humans speaking. The information voices convey is both linguistic (what has been said) and indexical (sociocultural status, emotional state, physical attributes, etc.; Giles & Powsland, 1975; Krause, Freyberg, & Morsella, 2002; Ladefoged & Broadbent, 1957; Murray & Arnott, 1993; Sachs, Lieberman & Erikson, 1972). This article concerns one of the most salient and important pieces of indexical information—whether someone speaking is a man or a woman. Of particular interest is how speaker-sex discrimination performance builds up with stimulus duration where the speech sounds are either voiced or whispered.

The communication sounds of mammals (including the speech sounds of humans) are produced by the same underlying physiological mechanism. The diaphragm pushes air from the lungs past the vocal folds. The vocal folds are muscular bands of tissue located in the larynx at the base of the throat. In normal *voiced* speech, the vocal folds open-and-close very rapidly in a vibratory motion which has the effect of breaking up the steady stream of air from the lungs into a series of discrete air puffs (glottal pulses). The number of these glottal pulses per second—the glottal-pulse rate (GPR)—determines the fundamental frequency (*f* 0) of the laryngeal source. The perceived pitch of the voice is highly correlated with *f* 0. However, in *whispered* speech, the vocal folds are held partially open (abducted) and do not vibrate. The steady stream of air from the lungs in whispered speech passes straight through the partially open glottis (space between the abducted vocal folds). Crucially, because of the lack of vibration in the vocal folds, no repetitive temporal structure is formed in the turbulent airflow of whispered speech. Whispered speech has thus no fundamental frequency and hence no temporal pitch associated with it.

For both voiced and whispered speech, after passing through the vocal folds, the glottal pulses (voiced) or broad-band noise (whispered) enter into the space above the larynx (the supralaryngeal vocal tract). For both types of speech, the frequency content entering into the vocal tract is differentially reinforced by the resonances of the vocal tract. The vocal tract resonances lead spectral prominences, known as formants, to form in the input frequency spectrum; with different formants distinguishing the different speech sounds (Peterson & Barney, 1952). The vocal tract resonances are determined by the configuration of the vocal tract which can be rapidly changed by different positioning of the various mobile articulators such as tongue, lips, jaws, and soft palate, and so forth. For the general principles of speech production, see Fant (1970) and Titze (2000).

The voices of men and women (and children) are distinguished by characteristic differences in GPR (Titze, 1989) and vocal-tract length (Fant, 1970; Fitch & Giedd, 1999). The length and mass of the vocal folds dictate the rate at which they can vibrate—the larger mass of a man’s vocal folds do not permit as rapid a vibration as those of a woman or child (Titze, 1989). Sexual dimorphism in GPR and hence *f*0 is marked, with men having a mean *f*0 of around 130 Hz and women having a mean *f*0 of 220 Hz (Hillenbrand, Getty, Clark & Wheeler, 1995). Such a difference is highly salient given that listeners can detect a 2% difference in voice pitch of individual vowels (Smith, Patterson, Turner, Kawahara, & Irino, 2005), thus *f*0 is a strong cue to speaker sex (e.g., Lass, Hughes, Bowyer, Waters, & Bourne, 1976; Whiteside, 1998).

The length of the supralarygneal vocal-tract is highly correlated with speaker height (Fitch & Giedd, 1999). As vocal-tract length (VTL) increases, the formants in speech shift toward lower frequencies (Fant, 1970). When we add the spurt in VTL arising from increased testosterone in pubertal male adolescents which stimulates growth in the laryngeal cartilages (Beckford, Rood, & Schaid, 1985), to the generally greater height of males compared with adult females, we find that the formant frequencies of adult males are about 15% less than those of adult females (Fitch & Giedd, 1999; Hillenbrand et al., 1995; Peterson & Barney, 1952). This means that formant frequency consequent upon differences in VTL is also a potential cue for speaker sex (e.g., Coleman, 1976; Ingemann, 1968; Schwartz & Rine, 1968).

Smith (2014) investigated the pattern of speaker-sex discrimination performance both as a function of stimulus duration and across different manipulations of *f*0 and formants. The results suggested that for very brief duration vowel sounds the listener uses VTL-related perceptual cues (frequencies of the formants) to distinguish men’s voices from women’s voices. However, at the point at which the percept is available, the listener switches to increasingly using GPR-related perceptual cues (voice pitch). The JND for VTL- and GPR-related perceptual cues are of the order of 8% and 2%, respectively (Smith et al., 2005). The suggestion is that in a speaker-sex discrimination task, the listener combines what information is available using early-available (but less reliable) information at the start of the decision process but, as time exposed to the stimulus increases, switches to late-available (but more reliable) information. Such an approach (which can be characterized as Bayesian) maximizes performance in a rapidly changing dynamic environment. This reflects a general philosophy of increasing the weighting of the more reliable cue when combining across multiple information sources (e.g., Hillis, Watt, Landy, & Banks, 2004; Jacobs, 2002) where the reliability of those cues change over time (for review of Bayesian learning see Knill & Pouget, 2004).

**Figure 1.**
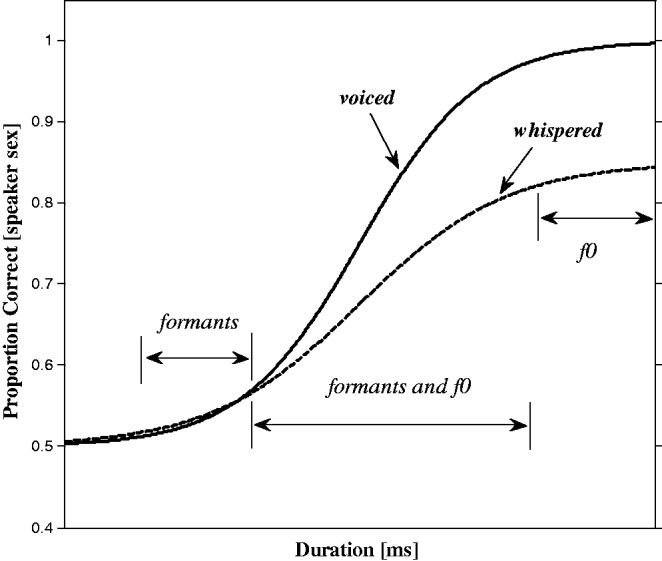
Hypothetical speaker-sex discrimination performance as a function of duration for voiced (solid line) and whispered (dashed line) speech. The general form of the psychometric functions is *P*(*t*) = γ + (1 – γ – λ)*F*(*t*), where *P*(*t*) is the probability of correct discrimination of speaker sex at stimulus duration *t*, with guess rate γ (which in an *m*AFC task is 1/*m,* or ½ in our 2AFC task) which sets the lower asymptote representing chance performance, and with lapse rate λ which sets the upper asymptote representing ceiling performance. The function *F* is for convenience taken to be the logistic function [1 + *exp*(−*x*)]^−1^, which takes values between 0 and 1 for values of *t*, −∞ < *t* < ∞ (see Treutwein & Strasburger, 1999). The bracketed region “formants” indicates durations where VTL-related information (the formants of speech) are the *main* cue to speaker sex discrimination, the region “*f*0” indicates durations where GPR-related information (voice pitch, as determined by *f*0) is the *main* cue for discriminating speaker sex, and the region “formants and *f*0” indicates durations where both formants and *f* 0 could contribute to speaker-sex discrimination. Proportion correct values on the *y* axis are for illustrative purposes only and *x*axis durations are purposively left blank.

One prediction of this view of how perceptual information is recruited across different time scales is that there should be different speaker-sex discrimination performance as a function of stimulus duration for *whispered* compared with *voiced* speech. When humans whisper, the normal vibratory motion of the vocal folds is suspended, and consequently there is no periodic *f*0 component in whispered speech. This contrasts with voiced speech, where the glottal pulses generated as the vocal folds vibrate, form a periodic *f*0 component in the speech sound which is clearly heard as the pitch of the voice. Thus, voiced speech has an extra speaker-sex cue of voice pitch compared with whispered speech. Interestingly, pitch needs at least two glottal cycles to be present in the sound, so for durations less than two glottal cycles both voiced and whispered speech possess no pitch information. However, both whispered and voiced speech have formant peaks imposed on their frequency spectrum by the filtering action of the vocal tract, so they both have VTL-related cues to speaker sex. Speaker-sex discrimination performance as a function of stimulus duration for whispered speech should thus take a different form than for voiced speech. At the very shortest of durations, where speaker-sex discrimination performance is driven by early-available VTL-related cues (Smith, 2014), voiced and whispered speaker-sex discrimination performance should be similar. But as stimulus duration increases and GPR-related information becomes available, voiced speech performance should improve relative to whispered speech performance (as shown in Figure 1). Thus, the underlying psychometric functions, which relate stimulus duration to listeners’ correct speaker-sex discrimination responses, are predicted to be markedly different for voiced and whispered speech.

## Method

### Participants

Twenty English-speaking listeners participated in the main experiment (14 female, age range 18–39 years, mean = 20.3 years). A different group of seven English-speaking listeners participated in the supplementary experiment (five female, age range 19–21 years, mean = 20.1 years). All listeners had normal hearing as indicated by their absolute thresholds at both ears at 0.5, 1, 2, and 4 kHz on an audiogram. Listeners were naive to the purpose of the experiments and participated to earn course credit. Written informed consent was given by the participants after the experiments were introduced to them. The experimental procedure was approved by the Hull Psychology Research Ethics Committee (Ref: 1415122506).

### Stimuli and Apparatus

Full details of the stimuli and procedures used in this study are given in Smith (2014) and will only be summarized here. One example of each of the five English vowels /a/, /e/, /i/, /o/, /u/, corresponding to the vowel sounds in “fa”, “bay”, “bee”, “toe,” and “zoo,” of four adult men and four adult women speakers were presented to listeners. Speakers provided both voiced and whispered versions of the vowels. The speakers were native-English speaking students at the University of Hull. Sounds were recorded with a sampling rate of 48 kHz and an amplitude resolution of 16-bits.

The duration of all vowels was adjusted to have six different durations (8, 12, 18, 27, 40, and 60 ms) by taking different duration length segments from the central portion of each vowel. Each segment was cosine-square gated to ensure that the sounds came on and went off smoothly over the first and last 1 ms, respectively. All the vowel sounds of all durations were normalized to the same root-mean-squared (rms) level of 0.0250 (relative to maximum of ± 1). The sound level of the vowels at the headphones was 77 dB SPL.

A noise mask was presented immediately following the offset of the short duration vowel. The Gaussian noise mask was 500 ms in duration, with an onset and offset that was smoothed by a cosine-gating function of 10 ms. The sound level of the Gaussian noise at the headphones was 69 dB SPL.

The stimuli were played by a 24-bit sound card (X-fi Xtreme Audio, Sound Blaster, Creative) and presented to the listener diotically over Sennheiser HD600 headphones. Listeners were seated in a single-walled IAC sound-attenuating booth.

### Procedure

The experiments were performed using a single-interval, one-response paradigm. The listener heard a vowel of a given duration and had to indicate whether a man or women had spoken the vowel. There was a 50% chance that either a man or woman had spoken the original vowel. There was a 20% chance that the vowel was a particular vowel from the set of five (/a–u/). The judgement of the sex of the speaker of the vowel uttered was made by selecting the appropriate button on a visual display. The order of the “man” and the “woman” buttons was quasi-randomly switched at the beginning of each run.

Listeners were first given a practice run of 30 trials with a single vowel duration of 100 ms of either voiced or whispered vowels. The purpose of the practice was to familiarize listeners with the experimental procedure. The five vowels were each presented six times, with half spoken by men and half spoken by women. Which vowel and whether the vowel was spoken by a man or a woman was quasi-randomly determined. The ability of listeners to correctly judge the sex of the original speaker was measured. Listeners invariably found it an easy task to judge the sex of the speaker of the voiced vowels at this duration (*M* = 99.17%, *SD* = 2.39% correct) but harder to judge the sex of the speaker of the whispered vowels (*M* = 83.50%, *SD* = 10.62% correct). Each listener was provided with feedback as to their performance level only for the first practice run (whether it was voiced or whispered being counterbalanced). The practice run took approximately 2 to 3 min to complete.

Listeners then proceeded on to the main experiment. The listener was given a run of 180 trials, consisting of six durations (8, 12, 18, 27, 40, and 60 ms), each repeated 30 times. Half the trials were vowels spoken by men, and half the trials were vowels spoken by women (balanced across durations and vowels). Each run consisted of either all voiced or all whispered vowels. The duration, sex, and vowel were presented in a quasi-random order generated by the computer. Which of the four men’s or four women’s vowels was used in any one trial was quasi-randomly determined by the computer. Whether listeners undertook the voiced-vowel run or the whispered-vowel run first was counterbalanced to control for the effects of experience or fatigue. There was no feedback. After the first experimental run had been completed, the listeners were given a practice run and then the last experimental run (all without feedback). Thus, one participant might do practice-voiced, experimental-voiced, practice-whispered then experimental-whispered. Another participant might do the whispered practice and experiment first, followed by the voiced practice and experimental conditions. Each experimental run of 180 trials took approximately 10 to 15 min to complete. Each listener did the experiment in one session lasting approximately 45 min.

## Results

**Figure 2.**
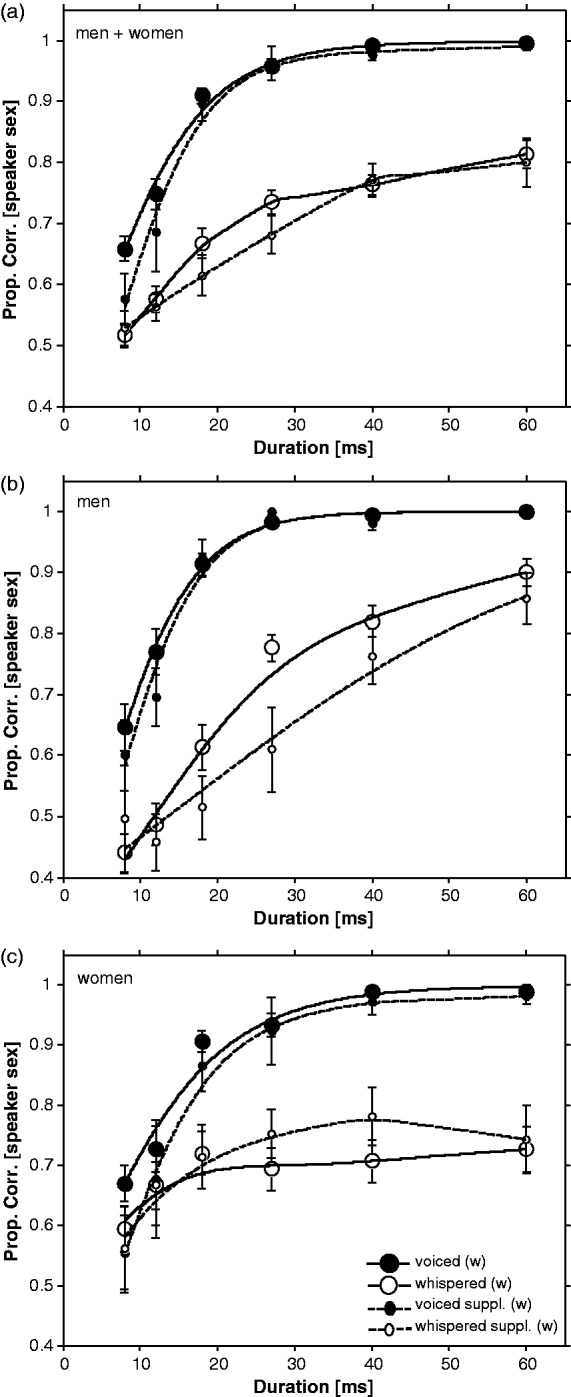
Proportion correct judgment of original speaker sex for voiced (filled circles) and whispered (open circles) vowels as a function of vowel duration. The large circles indicate the main experiment data. The small circles indicate the supplementary experiment data. The solid (fitted to main experiment data) and dashed (fitted to supplementary experiment data) curves are best-fitting psychometric functions using non-parametric local linear regression fitting (˙ychaluk & Foster, 2009). Data collapsed across correct judgments of both men and women speakers and across all five vowels (Figure 2(a), top). Data plotted separately for men speakers (Figure 2(b), middle) and women speakers (Figure 2(c), bottom). For the main experiment (Figure 2(a)), each point shown for each duration is based on 600 trials [(15 Men + 15 Women Speaker Repetitions) × 20 Listeners]. When plotted separately for the main experiment (Figure 2(b) and (c)), each datum point is based on 300 trials (15 Speaker Repetitions × 20 Listeners). The supplementary experiment data points are based on 210 trials [(15 Men + 15 Women Speaker Repetitions) × 7 Listeners] for Figure 2(a), and 105 trials (15 Speaker Repetitions × 7 Listeners) for Figure 2(b) and (c). Error bars are standard error of the mean across 20 listeners (main experiment) or 7 listeners (supplementary experiment).

Figure 2 shows proportion correct judgment of original speaker sex, as a function of duration of the vowel, for voiced and whispered vowels. The results for the main experiment (solid curves, large circles) are based on the mean data from 20 listeners, and the results for the supplementary experiment (dashed curves, small circles) are based on the mean data from 7 listeners. Results are presented pooled across both men and women speaker judgments (Figure 2(a), top), separately for men-speaker judgments (Figure 2(b), middle) and separately for women-speaker judgments (Figure 2(c), bottom).

### Main Experiment

The first finding is that proportion correct scores for the speaker-sex discrimination task are higher for voiced than for whispered vowels for all durations (Figure 2, solid curves, filled vs. open large circles). To characterize the relationship between vowel duration and proportion correct for the voiced and whispered vowels, an estimate of the psychometric function was made using non-parametric local linear regression fitting (Żychaluk & Foster, 2009). A “model-free”^1^ approach to estimating the psychometric function was adopted because the form of the underlying function is not known and because of the wish to avoid assumptions about lower and upper asymptote limits. The lower asymptote limit is conventionally set by the “guess rate” γ (which is 0.5 in a 2 Alternative Forced Choice (2AFC) task) and the upper asymptote limit is set by the maximum possible proportion correct minus the ‘lapse rate’ λ. Lapse rate represents incorrect responses that are unconnected to the level of the independent variable (due to momentary loss of attention and incorrect key presses) which tend to be minimal (affecting between 0% and 5% of trials, see Wichmann & Hill, 1999). However, “lapse” rate can be non-trivial if it incorporates a hard barrier to further improvements in performance, perhaps induced by lack of information, perceptual bias, change in the weighting, or cue used to make a decision. In parametric fitting of psychometric functions, both γ and λ substantially affect the shape of the fitting function (Treutwein & Strasburger, 1999; Wichmann & Hill, 2001). In our situation, there is no reason to assume that λ is trivially small or γ strictly equal to chance (0.5) because there may be perceptual biases or cue weighting changes affecting them. Local linear fitting derives the asymptotic values γ and λ automatically provided the psychometric function is sampled in the required region (Żychaluk & Foster, 2009). The non-parametric fits are as good as parametric fits, with the only assumption being that the function must be smooth (Żychaluk & Foster, 2009).

The point at which listeners can reliably tell whether a man or woman spoke—the duration threshold (*min_sex_*) for reliable discrimination, defined as the duration corresponding to the 0.75 point on the fitted curve (*d*′ = 1 for 2AFC task, see Macmillan & Creelman, 1991)—was extracted from the fitted psychometric functions to the voiced and whispered vowel conditions. The slope at a point equal to probability *P* = ((1 − γ − λ)/2 + γ) on the fitted curve was measured to provide a value for sensitivity—how quickly speaker-sex discrimination performance increases as a function of vowel duration. The reasoning was slope should not be unduly affected by differences in γ and λ which would arise if the slope was measured at a fixed probability such as 0.75.

The data were first analyzed pooled across both men’s and women’s voices (Figure 2(a)). The best-fitting psychometric function for voiced vowels (solid curve fitted to filled large circles) is clearly different from that of the whispered vowels (solid curve fitted to open large circles). The duration threshold (*min_sex_*) for reliable discrimination of whether a man or woman spoke was 11.28 (±0.45) ms for voiced vowels versus 33.77 (± 5.74) ms for whispered vowels. The uncertainty (*SD*) in the threshold and slope *M*s was estimated from 200 iterations in a bootstrap procedure (Foster & Bischof, 1991). Comparison of threshold (and slope) estimates across vowel types was made using 99% confidence intervals which maintain at least *p* < .01 for non-overlapping error bars when the standard error of the estimates differs by a factor of approximately 13 (see Payton, Greenstone, & Schenker, 2003). The threshold estimates for voiced and whispered vowels (Table 1) clearly do not overlap, and we can thus be confident that there is a significant difference between the duration thresholds for voiced and whispered vowels.

**Table 1.**
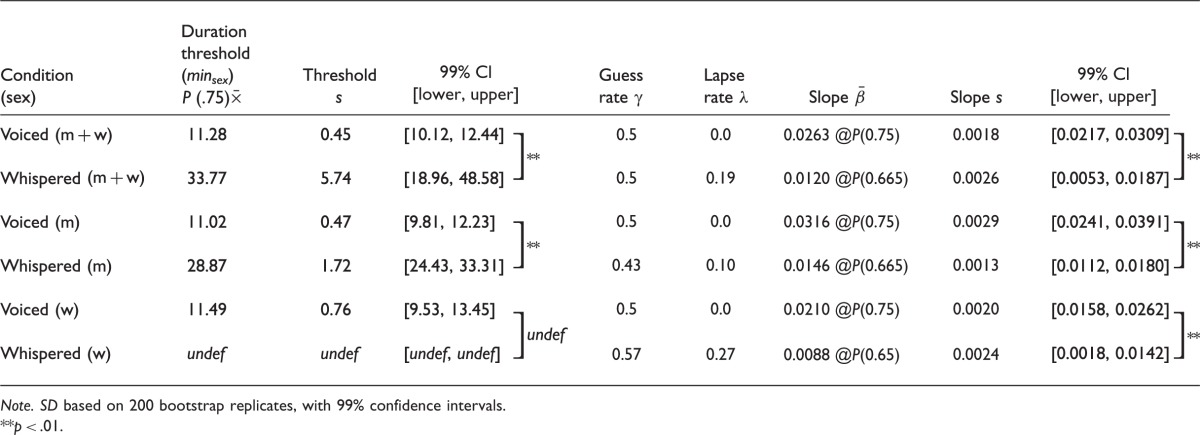
Mean Threshold (ms) and Slope Estimates Derived From the Best-Fitting Psychometric Functions for the Main Experiment.

*Note. SD* based on 200 bootstrap replicates, with 99% confidence intervals.

** *p* < .01.

A measure of the slope was also extracted from the fitted psychometric functions for the voiced and whispered vowels. These were measured at the *P* = .75 point for the voiced and at the *P* = .665 point for the whispered. The slopes were 0.0263 (± 0.0018) for the voiced and 0.0120 (± 0.0026) for the whispered, which are significantly different from each other at least at *p* < .01 (see Table 1).

The differences between voiced and whispered psychometric functions were also evident for the speaker-sex discrimination data for the men’s voices analyzed separately (Figure 2(b)). The threshold and slope estimates of the voiced and whispered vowels, derived from the fitted functions, clearly do not overlap—duration threshold (*min_sex_*) for reliably discriminating whether a man or woman spoke was 11.02 (± 0.47) ms for voiced vowels versus 28.87 (± 1.72) ms for whispered vowels, and slope estimates were 0.0316 (± 0.0029) for the voiced and 0.0146 (± 0.0013) for the whispered—all significantly different from each other at least at *p* < .01 (see Table 1).

Finally, differences between voiced and whispered psychometric functions were apparent when the speaker-sex discrimination data for the women’s voices were analyzed separately (Figure 2(c)). It is problematic to compare thresholds because the whispered condition for women’s voices *never* reaches 0.75 probability correct—however, clearly, there is a difference with duration threshold (*min_sex_*) for reliable discrimination whether a man or woman spoke being 11.49 (±0.76) ms for voiced vowels versus undefined (but at least >60 ms) for the whispered vowels. Comparing slope estimates, we have 0.021 (± 0.002) for the voiced and 0.0088 (± 0.0024) for the whispered, significantly different from each other at least at *p* < .01 (see Table 1).

### Supplementary Experiment

Although the voiced and whispered vowels were equated to the same level of 77 dB SPL, it could be argued that the whispered vowels are less salient than the voiced vowels. Thus, the reduced discriminability of speaker-sex in the whispered relative to the voiced vowels could be due to the whispered vowels having less perceptual loudness rather than their being impoverished in speaker-sex cues per se. To look at this idea further, the experiments were repeated but with the sounds all increased by 6 dB. All other details were the same.

Figure 2 (dotted line, small circles) shows probability correct judgment of original speaker sex, as a function of duration of the vowel, for voiced and whispered vowels in the supplementary experiment. As in the main experiment, the relationship between vowel duration and proportion correct for the voiced and whispered vowels, was characterized by using non-parametric local linear regression fitting (Żychaluk & Foster, 2009), to derive a best-fitting psychometric function. Threshold and slope estimates derived from the psychometric functions were compared between identical conditions across the supplementary and main experiment, for example, voiced (men and women speakers) in the supplementary versus voiced (men and women speakers) in the main experiment, whispered (men and women speakers) in the supplementary versus whispered (men and women speakers) in the main experiment, and so forth. In no case for the voiced vowels, was there a significant difference between comparable conditions in the main and supplementary experiments (compare following values against Table 1 equivalent values: voiced (m + w) *min_sex_* 13.10 (± 0.53) ms, slope 0.0308 (± 0.0032); voiced (m) *min_sex_* 12.16 (± 0.75) ms, slope 0.0343 (± 0.0055); voiced (w) *min_sex_* 14.19 (± 0.98) ms, slope 0.0256 (± 0.004)). For the whispered speaker-sex discrimination (men speakers), there was a significant difference between comparable conditions in the main and supplementary experiments (whispered (m) *min_sex_* 41.73 (± 3.36) ms, slope 0.0074 (± 0.0011)), while for the other whispered conditions there was no significant differences (whispered (m + w) *min_sex_* 36.68 (± 6.27) ms, slope 0.0065 (± 0.0029); whispered (w) *min_sex_* 56.36 (± 11.96) ms, slope 0.0019 (± 0.0042)).

## Discussion

This article investigated how speaker-sex discrimination performance improves as a function of stimulus duration for voiced and whispered vowels. The prediction was that speaker-sex discrimination performance for voiced and whispered vowels would be similar for very short durations but, as stimulus duration increased, voiced vowel performance would improve relative to whispered vowel performance. This would be reflected by markedly different psychometric functions (see hypothetical curves in Figure 1) and poorer speaker-sex discrimination performance (in terms of discrimination thresholds and sensitivity slope values) for whispered compared with voiced vowels. This is the case: a whispered vowel needs to have a duration three times longer than a voiced vowel before listeners can reliably tell whether it’s spoken by a man or woman (∼30 ms vs. ∼10 ms). Listeners are approximately half as sensitive to information about speaker-sex when it is carried by whispered as opposed to voiced vowels (as shown by the slopes of the psychometric functions).

It was suggested that the relative impairment between voiced and whispered speaker-sex discrimination performance should be least at shorter durations where the two different types of stimuli approach parity as both do not possess pitch information. This was partially confirmed (Figure 2(a), solid lines, filled vs. open large circles). Interestingly, when plotting judgments separately for men and women speakers the pattern of performance is more mixed. Men’s voices, though showing the characteristic poor speaker-sex discrimination performance of whispered vowels relative to voiced vowels, do not show *less* impairment at very short durations relative to longer durations (Figure 2(b)). Women’s voices are more similar to the prediction, showing little difference between whispered and vowel speaker-sex discrimination performance at short durations but a large difference at longer vowel durations (Figure 2(c)). Whispered speech tends to have higher formants (primarily *F*1) than voiced speech for a given vowel (Kallail & Emanuel, 1984). Higher-frequency formants cue for shorter VTL (Fant, 1970) which would indicate a women speaker, as women on average have shorter VTLs than men (Fitch & Giedd, 1989). This could lead to some misclassification of male vowels as being spoken by a woman. This seems to be occurring at least at very short durations (8 ms) where performance drops below chance (0.50) for men’s vowels (Figure 2(b)).

Another difference between speaker-sex discrimination performance for men’s and women’s whispered vowels is at longer durations (≥40 ms) where women’s whispered vowel speaker-sex discrimination performance asymptotes at approximately 0.70 proportion correct while men’s whispered vowel speaker-sex discrimination performance increases up to 0.90 (at 60 ms). The male glottis has a medial surface bulge in the vocal folds while the female glottis converges more linearly (Titze, 1989). This could lead to perceptual differences at longer durations in male and female whispered vowels which might aid speaker-sex discrimination. The difficulties associated with identifying women compared with men speakers is consistent with other studies that have shown a perceptual advantage for male sounds in speaker-sex discrimination tasks (Owren, Berkowitz, & Bachorowski, 2007).

A supplementary experiment exploring whether differences in loudness between voiced and whispered vowels might explain the observed pattern of speaker-sex discrimination performance involved increasing the sound level of the stimuli. This had no effect on performance for voiced vowels (Figure 2(a)–(c), small vs. large filled circles). The effect upon whispered vowels was only significant for men’s voices (Figure 2(b), small vs. large open circles), where increasing the sound level by 6 dB led to *poorer* performance at medium durations. There was no difference in speaker-sex discrimination performance for whispered women’s voices (Figure 2(c)) or when men’s and women’s voices were plotted together (Figure 2(a)). This implies that changes in perceptual loudness do not underlie differences in speaker-sex discrimination performance between voiced and whispered vowels. The suggestion is that the differences are due to a lack of temporal pitch information in whispered speech.

In summary, the impoverished representation of speaker-sex cues (no temporal pitch) in whispered speech leads to poorer speaker-sex discrimination performance for whispered compared with voiced vowels—a whispered vowel has to be three times as long (34 ms) as a voiced vowel (11 ms) to reach the threshold of discrimination (*min_sex_*). The difference between voiced and whispered vowel speaker-sex discrimination performance is *least* at very short durations because both voiced and whispered vowels contain VTL-related information *and* have no GPR-related information. However, at longer durations, GPR-related information becomes available in the voiced vowels while still being absent from the whispered vowels. Consequently, whispered vowel speaker-sex discrimination performance does not improve as much as voiced vowel speaker-sex discrimination performance. This is consistent with Smith (2014) in that it provides further support for the idea that speaker-sex discrimination is mediated by VTL-related information at the very shortest durations and then switches to being dominated by GPR-related information when it is available at longer durations. This makes best use of what information is available—using early-available but less reliable information in the beginning of a decision process and then switching to late-available but reliable information as it comes on stream. Such an approach maximizes performance in a rapidly changing dynamic environment.

## Note

1 No assumptions were made as to the underlying psychometric function model such as whether it was Weibull, Logistic, Gaussian, and so forth.
